# The association between dietary insulin index and load with mental health

**DOI:** 10.1186/s40359-022-00925-2

**Published:** 2022-09-19

**Authors:** Mina Darand, Ali Amirinejad, Amin Salehi-Abargouei, Ian G. Davies, Masoud Mirzaei, Mohsen Mazidi, Sayyed Saeid Khayyatzadeh

**Affiliations:** 1 Department of Clinical Nutrition, School of Nutrition and Food Science, Food Security Research Center, Isfahan University of Medical Sciences, Isfahan, Iran; 2 Nutrition and Food Security Research Center, Shahid Sadoughi University of Medical Sciences, Shohadaye Gomnam BLD. ALEM Square, Yazd, Iran; 3 Department of Nutrition, School of Public Health, Shahid Sadoughi University of Medical Sciences, Yazd, Iran; 4 Research Institute of Sport and Exercise Science, Liverpool John Moores University, Liverpool, UK; 5 Yazd Cardiovascular Research Center, Shahid Sadoughi University of Medical Sciences, Yazd, Iran; 6 Nuffield Department of Population Health, University of Oxford, Richard Doll Building, Old Road Campus, Oxford, OX3 7LF UK

**Keywords:** Anxiety, Depression, Dietary insulin index, Dietary insulin load, Stress

## Abstract

**Background:**

Depression, anxiety, and stress are common mental problems. The aim of this cross-sectional study was to investigate the association between two indexes that measure postprandial insulin response to different food, dietary insulin index (DII) and insulin load (DIL), with psychological disorders.

**Method:**

Participants (n = 10,000) aged 20–69 were randomly selected from 200 clusters in Yazd from the recruitment phase of the Yazd Health Study. The dietary intake of participants was collected by a reliable and validated food frequency questionnaire (FFQ) consisting of 178 food items. DII and DIL were calculated from the FFQ data using previously published reference values. To assess psychological disorders an Iranian validated short version of a self-reported questionnaire (Depression Anxiety Stress Scales 21 [DASS21]) was used.

**Results:**

No significant association was observed between DIL and DII with odds of depression or anxiety using crude or adjusted models. However, individuals in the highest quartiles of DIL had the lowest odds of stress (OR: 0.69; 95% CI 0.48–1.01, *P*-trend = 0.047). This association remained significant after adjustment for potential confounders in model II including marital status, smoking, education, job status, salt intake, and multi-vitamin supplement use (OR: 0.38; 95% CI 0.16–0.91, *P*-trend = 0.039) and the third and final model which is further adjusted for BMI (OR: 0.39; 95% CI 0.16–0.91, *P*-trend = 0.041).

**Conclusion:**

Overall, consumption of foods with higher DII as well as DIL were associated with lower stress scores; however, no significant relationship was observed between DII or DIL with respective depression or anxiety scores.

**Supplementary Information:**

The online version contains supplementary material available at 10.1186/s40359-022-00925-2.

## Introduction

Depression, anxiety, and stress are major mental health concerns in most populations. Recent reports of the global prevalence of depression, anxiety, and distress show 31.4%, 31.9% and 41.1% respectively, which is much higher than normal levels due to the COVID-19 pandemic [1]. Poor mental health imposes considerable costs on international health systems. A wealth of evidence shows psychological distress is associated with disability, early mortality, and increased onset of chronic disease such as metabolic syndrome, diabetes, cardiovascular disease, and Alzheimer’s disease [2–5].

The prevalence of depression, anxiety, and stress is much higher in diabetic patients compared to those without, and the role of insulin resistance and hyperinsulinemia in the progression of mental disease has been well-established [6–8].

Diet has a considerable effect on serum insulin levels and a high glycemic index (GI) and high glycemic load (GL) diet may be related to the higher risk of depression [9]. GI and GL indicate the ability of foods to raise blood glucose levels compared to glucose or white bread [10]. While high serum glucose levels correlates with insulin levels, foods containing certain amino acids and fatty acids can induce insulin secretion with a dampened effect on glucose levels [11–13]. An insulin index (II) has been suggested to estimate postprandial insulin levels in response to isoenergetic amounts of different foods [14]. The dietary insulin index (DII) and dietary insulin load (DIL) can be calculated by considering the DII of each food by its energy content and the frequency of consumption in the diet [15]. Because the DII indicates insulin responses directly it is arguably a better predictor of disease related to insulin resistance compared to GI.

Some previous studies have shown that higher DII and DIL may be associated with increased risk of obesity and insulin resistance [16, 17]. The negative effects of obesity on psychological disorders have been observed in several studies [18, 19]. Mechanisms include hypertrophy of adipose tissue which is accompanied by an inflammatory response that can lead to neuroinflammation [20, 21].

To our knowledge, recently, only one study investigated the relationship between DII and DIL with psychological disorders in the 3172 Iranian adults. The results of this study indicated that higher DII and DIL were related with greater odds of depression in women. However no significant association was observed between DII, DIL and anxiety [22]. Considering the role of hyperinsulinemia in psychological disorders and the necessity of more studies with larger sample sizes, we investigated this hypothesis in 7384 Iranian adults.

## Materials and methods

### Study population

In the current study, we used data from a Yazd Health Study (YaHS) conducted from September 2014 to December 2015 in Yazd, in central Iran. YaHS is a prospective cohort study, which includes n = 10,000 participants aged 20–69 y who were randomly selected for the recruitment phase from 200 clusters in Yazd Greater Area. The profile and details of this study were published elsewhere [23]. Written informed consent was obtained from all participants. The research was approved by the Ethics Committee of Shahid Sadoughi University of Medical Sciences, Yazd, Iran (Ethic code: 931188). Data about current and history of chronic diseases, smoking status, and socio-demographic characteristics including age, gender, marriage status and education level were obtained by interview and standard questionnaires. Participants were excluded on the following: under or over estimation of energy intake (total daily energy intake < 800 or > 6500 kcal/day), pregnancy, following a special diet and taking antidepressants. After exclusion, n = 7574 participants participated in the study.

### Dietary assessment

Dietary intake of study participants was collected by a reliable and validated food frequency questionnaire (FFQ) consisting of 178 food items designed for an Iranian population [24]. FFQs were completed during face-to-face interviews and reported dietary intakes in household measures were converted to grams and inputted into the Nutritionist IV software (First Databank Inc., Hearst Corp., San Bruno, CA, USA). To calculate daily nutrient intake values for each participant, the US Department of Agriculture’s (USDA) national nutrient databank was used [25].

### Dietary insulin index and load calculation

Briefly, the food insulin index (FII) refers to the incremental insulin area under the curve over a 2 h period after consumption of a 1000-kJ (239 kcal) portion of the test food divided by the area under the curve after ingestion of a 1000-kJ portion of the reference food. The insulin indices for 61 food items were obtained from previous publications [26–28]. For the remaining 71 food items that were not available in the published food lists, the FII of similar food items was used by the correlation between their energy, fiber, carbohydrate, protein, and fat content.

To determine the DIL, we first calculated the insulin load of each food with the following formula:

Insulin load of food = Insulin index of food × energy content of food (kcal**/**d)

Secondly, by summing up the insulin load of each food, the DIL was obtained. We calculated the average DII for all foods by dividing DIL by total dietary intake of energy.

### Anthropometric measurements

Body weight was measured using a portable digital scale analyser with an accuracy of 0.1 kg. The participants stood in the middle of the scale, wearing the minimum possible clothing. Height was also measured in a standing position, while barefoot with the head placed in the Frankfurt position. Body Mass Index (BMI) was calculated, body weight (kg)/ height (m^2^). All anthropometric measurements were performed before starting the interview, again after having completed one-third of the questionnaire and for a final time after completing two-thirds of the questions in the questionnaire.

### Psychological health assessment

An Iranian validated short version of the self-report questionnaire for depression, anxiety and stress (DASS 21) consisting of seven items per subscale was used [29]. The individuals read each statement and recorded their reply according to a 4-point Likert scale ranging from 0 (Does not apply to me at all) to 3 (Applies to me very much or most of the time). The scores were summed for items of each scale. As the long form of DASS has 42 items, we multiplied the final score of each scale by two. The individuals were considered to have depression, anxiety, and stress if they obtained total scores of ≥ 10, ≥ 8 and ≥ 15, respectively [30].

### Statistical analysis

The normality of data was assessed using the Kolmogorov–Smirnov test. Continuous and categorical variables were compared across quartiles of DIL and DII using analysis of variance (ANOVA) and chi-square tests respectively. post-hoc analysis was performed using post-hoc Bonferroni tests. Logistic regression was applied to evaluate the relationship between DII and DIL with psychological disorders in crude and adjusted models. In Model I basic information were adjusted. In Model II, socioeconomic status and behavioural risk factors were additionally controlled. More adjustment was performed for chronic condition. Model I was adjusted for age, gender, and total energy intake. In model II, marital status, smoking, education level, employment status, salt intake and multi-vitamin use were additionally adjusted. The final model included the addition of BMI. These confounding variables were included in the model because previous studies showed associations with the psychological disorders. *P*-values < 0.05 were considered statistically significant. To analyze the data, the statistical Package for Social Sciences (SPSS) (version 23.0, SPSS Inc., Chicago, Illinois, USA) was used.

## Results

The general characteristics of study participants across quartiles of DIL and DII are indicated in Table 1 and Additional file 1: Table S1. The final sample size included in our analyses was n = 7574 participants. The prevalence of psychological disorders did not significantly differ between quartiles of DIL and DII, except for anxiety prevalence, which was greater in the highest quartile of DII. Significant differences were observed for BMI, age, marital status, gender, employment status, education level and multi-vitamin supplement use across quartiles of DIL. These significant differences were only obtained for employment status, education level, and multi-vitamin supplement use among quartiles of DII. The distribution of smoking status did not differ throughout quartiles of DII and DIL. In addition, post-hoc analysis was performed by Bonferroni post-hoc test. Significant differences among the quartiles of DIL and DII are shown with different letters “a” or “b”.

**Table 1:** General characteristics of study participants across quartiles of DIL and DII

Variable	Dietary insulin load						Dietary insulin index				
	Total	Q1 (n = 1893)	Q2 (n = 1894)	Q3 (n = 1894)	Q4 (n = 1893)	*P*-value ^I^	Q1 (1893)	Q2 (1894)	Q3 (1894)	Q4 (1893)	*P*-value
Depression, yes (%)	578 (8.1)	154 (8.6)	157 (8.8)	138 (7.6)	129 (7.3)	0.268	149 (8.3)	146 (8.1)	120 (6.7)	163 (9.2)	0.057
Anxiety, yes (%)	754 (10.5)	196 (11)	195 (10.9)	188 (10.3)	175 (9.9)	0.685	199 (11.1)	180 (9.9)	164 (9.2)	211 (11.8)	0.041
Stress, yes (%)	238 (3.3)	70 (3.9)	62 (3.5)	57 (3.1)	49 (2.8)	0.264	64 (3.6)	64 (3.5)	49 (2.7)	61 (3.4)	0.456
BMI (kg/m2)	27 ± 5.09^II^	27.28 ± 5.01	26.82 ± 5.23	26.92 ± 5.06	26.97 ± 5.05	0.036	27.22 ± 5.10	26.83 ± 5.00	26.93 ± 5.08	27.01 ± 5.18	0.12
Age (%)											
20–29 years	1586 (21.5)	318 (17.2)^a^	404 (21.9)^b^	421 (22.6)^b^	443 (24.1)^b^	< 0.01	395 (21.6)	398 (21.4)	418 (22.4)	395 (21.4)	0.499
30–39 years	1598 (21.6)	310 (16.8)^a^	402 (21.8)^b^	448 (24.1)^b^	438 (23.8)^b^		382 (20.9)	408 (21.9)	413 (22.4)	395 (21.4)	
40–49 years	1586 (21.5)	402 (21.8)	409 (22.2)	388 (20.8)	387 (21.1)		408 (22.3)	398 (21.4)	395 (21.4)	385 (20.9)	
50–59 years	1402 (19)	377 (20.4)	350 (19)	340 (18.3)	335 (18.2)		347 (19)	367 (19.7)	314 (17)	374 (20.3)	
60–69 years	1215 (16.4)	438 (23.7)^a^	277 (15)^b^	265 (14.2)^b^	235 (12. 8)^b^		299 (16.3)	293 (15.7)	307 (16.6)	316 (17.1)	
Marriage (%)											
Single	856 (11.6)	171 (9.3)^a^	227 (12.4)^b^	228 (12.2)^b^	230 (12.5)^b^	< 0.01	227 (12.4)	224 (12.1)	212 (11.5)	193 (10.5)	0.505
Married	6264 (85.1)	1575 (85.6)	1547 (84.8)	1588 (85.3)	1554 (84.8)		1539 (84.1)	1570 (84.6)	1575 (85.6)	1580 (86)	
Widowed or Divorced	240 (3.3)	95 (5.2)^a^	50 (2.7)^b^	46 (2.5)^b^	49 (2.7)^b^		63 (3.4)	61 (3.3)	52(2.8)	64 (3.5)	
Smoking status (%)											
Never smoker	6327 (87.8)	1560 (87.3)	1604 (88.5)	1564 (87.6)	1578 (87.8)	0.796	1598 (88.2)	1578 (87.2)	1601 (88.1)	1551 (87.7)	0.708
Current smoker	766 (10.6)	204 (11.4)	178 (9.8)	192 (10.6)	192 (10.7)		189 (10.4)	197 (10.9)	187 (10.3)	193 (10.9)	
Ex_smoker	113 (1.6)	23 (1.3)	30 (17)	32 (1.8)	28 (1.6)		25 (1.4)	35 (1.9)	29 (1.6)	24 (1.4)	
Gender, male (%)	3673 (49.7)	809 (43.8)^a^	931 (50.6)^b^	983 (51.7)^b^	950 (51.9)^b^	< 0.01	899 (48.9)	910 (48.9)	917 (49.7)	947 (51.4)	0.388
Employment (%)											
Unemployed	1415 (19.5)	363 (20.2)	343 (19)^a^	324 (17.7)^b^	385 (21.3)	< 0.01	1415 (19.5)	3537 (48.8)^b^	247 (3.4)^b^	2046 (28.2)^a^	0.015
Government employee	3537 (48.8)	976 (54.2)^a^	895 (49.5)^b^	867 (47.5)^b,c^	799 (44.1)^c^		348 (19.4)^b^	392 (18)^b^	330 (18.2)	408 (22.5)^a^	
Manual worker	247 (3.4)	60 (3.3)	52 (2.9)	75 (4.1)	60 (3.3)		898 (50.1)	920 (50.4)	897 (49.4)	822 (45.4)	
Freelance job	2046 (28.2)	401 (22.3)^a^	518 (28.7)^b^	561 (30.7)^b^	566 (31.3)^b^		58 (3.2)	68 (3.7)	62 (3.4)	59 (3.3)	
Education (%)											
Illiterate	1776 (24.1)	577 (31.4)^a^	385 (20.9)^b^	412 (22.1)^b^	402 (22.1)^b^	< 0.01	439 (23.9)	471 (25.4)	421 (23)	445 (24.2)	0.01
Middle school	2095 (28.5)	503 (27.4)	552 (30)	508 (27.3)	532 (29.2)		490 (26.7)^b^	495 (26.7)^b^	514 (28)^b^	596 (32.4)^a^	
Diploma	2289 (31.1)^a^	527 (28.7)	566 (30.8)^b^	615 (33)	581 (31.9)		589 (32.1)	563 (30.4)	593 (32.3)	544 (29.6)	
Bachelor’s degree	995 (13.5)	195 (10.6)^a^	270 (14.7)^b^	266 (14.3)^b^	264 (14.5)^b^		256 (14)	271 (14.6)	253 (13.8)	215 (11.7)	
Master and doctor	205 (2.8)	35 (1.9)^a^	65 (3.5)^b^	61 (3.3)	44 (2.4)		60 (3.3)	55 (3)	53 (2.9)	37 (2)	
Multi*-*vitamins supplement (%)											
Never	6407 (88.2)	1722 (94.3)^a^	1727 (94.2)^a^	1666 (91)^b^	1292 (72.7)^c^	< 0.01	1549 (85.2)^a^	1616 (88.9)^b^	1607 (89.8)^b^	1635 (88.8)^b^	< 0.01
1–3/month	482 (6.6)	41 (2.2)^a^	46 (2.5)	74 (4)^b^	321 (18.1)^c^		147 (8.1)^a^	103 (5.7)^bc^	94 (5.3)^c^	138 (7.5)^ab^	
Minimal once a week	378 (5.2)	63 (3.5)^a^	60 (3.3)^a^	90 (4.9)^b^	165 (9.3)^c^		123 (6.8)^a^	99 (5.4)^a^	88 (4.9)^a^	68 (3.7)^b^	
Energy intake (kcal)	2897 ± 1378	1580 ± 298^a^	2161 ± 376^b^	1318 ± 569^b^	4828 ± 1015^b^	0.001 <	3207 ± 1394^a^	2822 ± 1306^b^	2658 ± 1301^b^	2900 ± 1449^b^	0.001 <

Those values which are indicated with different superscripts (a, b and c) have significant differences (*P*-value < 0.05)

BMI, body mass index

^I^Obtained from χ2 test and One-way Anova for categorical and continuous variables, respectively

^II^Data are presented as mean ± standard deviation (SD)

^a,b,c^post-hoc analysis was performed by Bonferroni post-hoc test

Dietary nutrients and energy adjusted food groups are shown in Table 2 and Additional file 2: Table S2. The one-way ANOVA test followed by Bonferroni post-hoc analysis revealed significant differences between all dietary nutrients and food groups including, energy intake; percentage energy from protein, carbohydrate, and fat intake; cholesterol; saturated fatty acid; vitamin E; vitamin C; folic acid; magnesium; fruits; vegetables; red meat; fish, dairy; whole grains; refined grains; sugars; salt; legumes and nuts. Significant differences among the quartiles of DGI and DGL are shown with different letters “a” or “b”.

**Table 2:** Dietary intakes of study participants across quartiles of dietary insulin index and load

Dietary insulin load							Dietary insulin index				
	Total	Q1	Q2	Q3	Q4	*P-*value^I^	Q1	Q2	Q3	Q4	*P-*value
*Nutrients*											
Energy intake (kcal)	2897 ± 1378^II^	1580 ± 298^a^	2161 ± 376^b^	1318 ± 569^b^	4828 ± 1015^b^	<0.001	3207 ± 1394^a^	2822 ± 1306^b^	2658 ± 1301^b^	2900 ± 1449^b^	<0.001 >
Protein (% of total daily energy)	0.16 ± 0.04	0.17 ± 0.03^a^	0.16 ± 0.04^b^	0.14 ± 0.04^b^	0.16 ± 0.04^b^	<0.001	0.17 ± 0.05^a^	0.16 ± 0.03^b^	0.15 ± 0.03^b^	0.14 ± 0.03^b^	<0.001 >
Carbohydrate (% of total daily energy)	0.54 ± 0.08	0.55 ± 0.08^a^	0.55 ± 0.09	0.58 ± 0.11	0.56 ± 0.09^b^	<0.001	0.48 ± 0.09^a^	0.55 ± 0.07^b^	0.57 ± 0.07^b^	0.62 ± 0.08^b^	<0.001
Fat (% of total daily energy)	0.35 ± 0.10	0.32 ± 0.07^a^	031 ± 0.07^b^	0.34 ± 0.08^b^	0.35 ± 0.10^b^	<0.001	0.39 ± 0.09^a^	0.33 ± 0.07^b^	0.31 ± 0.07^b^	0.29 ± 0.08^b^	<0.001
Cholesterol (mg)	394.8 ± 391.2	242.7 ± 108.4^a^	323.3 ± 290.5^b^	433.3 ± 350.5^b^	580.1 ± 574.1^b^	<0.001	522.8 ± 567.6^a^	395.9 ± 310.8^b^	345.7 ± 252.2^b^	315.04 ± 324.4^b^	<0.001
SFA (g)	31.04 ± 18.36	17.87 ± 6.08^a^	23.51 ± 8.23^b^	33.16 ± 13.52^b^	49.64 ± 21.93^b^	<0.001	39.22 ± 22.34^a^	30.54 ± 16.02^b^	27.52 ± 15.34^b^	26.90 ± 16.15^b^	<0.001
Vitamin E (mg/day)	11.36 ± 11.66	7.14 ± 5.71^a^	9.23 ± 7.85^b^	12.73 ± 12.95^b^	16.34 ± 15.27^b^	<0.001	16.16 ± 17.91^a^	11.0 ± 9.15^b^	9.83 ± 7.90^b^	8.45 ± 6.59^b^	<0.001
Vitamin C (mg)	210.5 ± 188.7	115.1 ± 59.8^a^	163.03 ± 89.7	236.09 ± 145.5^b^	327.1 ± 289.9^b^	<0.001	225.7 ± 171.1^a^	224.3 ± 199.9	197.2 ± 173.1^b^	195.03 ± 206.07^b^	<0.001
Folic acid (µg)	379.8 ± 228	220.68 ± 77.38^a^	297.9 ± 127.06^b^	414.1 ± 183.9^b^	586.75 ± 275.4^b^	<0.001	447.9 ± 264.7^a^	387.9 ± 233^b^	394.5 ± 197.1^b^	334.02 ± 192.5^b^	<0.001
Magnesium (mg)	193.4 ± 52.2	193.4 ± 52.2^a^	285.7 ± 75^b^	361.2 ± 112.7^b^	543.9 ± 194.80^b^	<0.001	388.7 ± 197.07^a^	340.4 ± 171.8^b^	312.3 ± 165.4^b^	315.9 ± 172.4^b^	<0.001
*Food groups (g/1000 kcal)*											
Fruits	184.3 ± 138.4	193.8 ± 111.5^a^	197.1 ± 128.8^b^	194.3 ± 141.2^b^	151.8 ± 162.09^b^	<0.001	165.6 ± 113^a^	185.02 ± 125.4^b^	189.1 ± 128.1^b^	197.4 ± 176.6^b^	0.001
Vegetables	96.6 ± 77.2	104.6 ± 69.8^a^	97.46 ± 82.99	86.65 ± 69.25^b^	96.68 ± 77.29^b^	<0.001	107.07 ± 98.2^a^	100.6 ± 66.6	98.03 ± 74.16^b^	80.99 ± 62.76^b^	0.001
Red meat	20.46 ± 18.37	22.41 ± 15.65^a^	22.46 ± 14.61	22.47 ± 21.33	14.49 ± 19.74^b^	<0.001	20.87 ± 20.36^a^	21.78 ± 18.49	21.09 ± 17.56	18.09 ± 16.67^b^	0.001
Fish	7.00 ± 13.56	5.60 ± 6.64^a^	6.02 ± 11.62	7.50 ± 14.90	8.79 ± 18.13^b^	<0.001	7.58 ± 14.64^a^	7.80 ± 13.06	7.53 ± 16.64	5.07 ± 8.22^b^	0.001
Dairy	89.84 ± 60.15	104.89 ± 54.36^a^	95.01 ± 53.72	86.57 ± 61.00^b^	72.88 ± 66.10^b^	<0.001	98.17 ± 80.10^a^	94.38 ± 55.93	91.45 ± 50.61^b^	75.35 ± 45.55^b^	0.001
Legumes and nuts	25.20 ± 19.14	26.40 ± 15.25^a^	25.37 ± 18.19^b^	26.21 ± 22.49^b^	22.82 ± 19.73^b^	<0.001	29.15 ± 24.82^a^	25.23 ± 17.59^b^	24.43 ± 16.13^b^	21.98 ± 15.95^b^	0.001
Whole grains	29.95 ± 27.33	35.96 ± 27.37^a^	37.26 ± 27.87^b^	28.17 ± 27.47^b^	18.41 ± 21.99^b^	0.001	22.63 ± 24.35^a^	28.14 ± 25.99^b^	32.12 ± 24.91^b^	36.93 ± 31.47^b^	0.001
Refined grains	85.67 ± 58.18	100.4 ± 55.67^a^	102.7 ± 50.99^b^	79.7 ± 52.7^b^	59.69 ± 62.02^b^	<0.001	71.67 ± 47.77^a^	87.49 ± 55.64^b^	96.35 ± 57.31^b^	85.67 ± 58.18^b^	0.001
Sugars	12.63 ± 11.64	12.25 ± 9.83^a^	12.13 ± 8.79^b^	12.70 ± 11.94^b^	13.45 ± 14.99^b^	<0.001	7.12 ± 6.25^a^	9.84 ± 7.35^b^	12.87 ± 8.70^b^	20.61 ± 16.51^b^	0.001
Salt	2.94 ± 3.68	4.17 ± .79^a^	3.56 ± 3.20	2.30 ± 3.30^b^	1.75 ± 3.86^b^	<0.001	2.59 ± 3.34^a^	2.87 ± 3.39	3.31 ± 3.90^b^	3.01 ± 4.00^b^	0.001

Those values which are indicated with different superscripts (a and b) have significant differences (*P*-value < 0.05)

DII, Dietary insulin index; DIL, Dietary insulin load

^I^Obtained from one-way Anova

^II^Data are presented as mean ± standard deviation (SD)

^a,b^post-hoc analysis was performed by Bonferroni post-hoc test

The associations between DIL and DII with the prevalence of depression, anxiety, and stress in crude and adjusted models are presented in Table 3. There were no significant associations between DIL and DII with odds of depression or anxiety in crude and adjusted models. However, individuals in the highest quartiles of DIL had the lowest odds of stress (OR: 0.69; 95% CI 0.48–1.01, *P*-trend = 0.047). This association remained significant after adjustment for potential confounding variables in model II (OR: 0.38; 95% CI 0.16–0.91, *P*-trend = 0.039) and model III (OR: 0.39; 95% CI 0.16–0.91, *P*-trend = 0.041).

**Table 3:** Multivariable-adjusted ORs (and 95% CIs) for depression, anxiety and stress across quartiles of dietary insulin index and load

Dietary insulin index						Dietary insulin load				
	Q1	Q2	Q3	Q4	*P*-trend^‡^	Q1	Q2	Q3	Q4	*P*-trend
*Depression*										
Crude	1	1.02 (0.81_1.29) ^†^	0.87 (0.68_1.10)	0.83 (0.65_1.06)	0.073	1	0.96 (0.75_1.22)	0.78 (0.61_1.01)	1.10 (0.87_1.39)	0.717
Model I*	1	1.04 (0.81_1.33)	0.88 (0.64_1.21)	0.86 (0.51_1.44)	0.491	1	0.93 (0.73_1.19)	0.75 (0.58_0.96)	1.08 (0.85_1.37)	0.846
Model II**	1	1.02 (0.79_1.33)	0.86 (0.61_1.20)	0.78(0.45_1.35)	0.375	1	0.94 (0.77_1.21)	0.74 (0.57_0.97)	1.05 (0.82_1.35)	0.944
Model III***	1	1.03 (0.79_1.34)	0.86 (0.62_1.21)	0.79 (0.45_1.37)	0.399	1	0.95 (0.74_1.22)	0.75 (0.57_0.98)	1.06 (0.83_1.36)	0.972
*Anxiety*										
Crude	1	0.99 (0.80_1.22)	0.93 (0.75_1.15)	0.89 (0.71_1.10)	0.242	1	0.87 (0.71_1.08)	0.80 (0.64_0.99)	1.07 (0.87_1.31)	0.686
Model I*	1	1.00 (0.80_1.25)	0.91 (0.69_1.20)	0.84 (0.53_1.33)	0.479	1	0.87 (0.70_1.07)	0.78 (0.62_0.97)	1.05 (0.85_1.30)	0.781
Model II**	1	0.96 (0.76_1.22)	0.86 (0.64_1.15)	0.73 (0.45_1.19)	0.251	1	0.88 (0.71_1.10)	0.77 (0.61_0.97)	1.01 (0.81_1.26)	0.847
Model III***	1	0.97 (0.76_1.22)	0.86 (0.64_1.15)	0.74 (0.46_1.21)	0.27	1	0.89 (0.71_1.11)	0.77 (0.61_0.98)	1.02 (0.81_1.26)	0.877
*Stress*										
Crude	1	0.88 (0.62_1.25)	0.79 (0.55_1.13)	0.69 (0.48_1.01)	0.047	1	0.98 (0.69_1.40)	0.75 (0.51_1.10)	0.95 (0.66_1.36)	0.499
Model I*	1	0.83 (0.57_1.21)	0.65 (0.40_1.05)	0.45 (0.20_1.01)	0.058	1	0.95 (0.67_1.36)	0.69 (0.47_1.02)	0.93 (0.65_1.34)	0.417
Model II**	1	0.83 (0.56_1.23)	0.62 (0.37_1.02)	0.38 (0.16_0.91)	0.039	1	0.98 (0.68_1.42)	0.72 (0.48_1.08)	0.88 (0.60_1.29)	0.284
Model III***	1	0.83 (0.56_1.24)	0.62 (0.37_1.03)	0.39 (0.16_0.91)	0.041	1	0.98 (0.68_1.42)	0.72 (0.48_1.08)	0.88 (0.60_1.29)	0.288

†These values are odds ratios (95% CIs)

‡Obtained from multivariate logistic regression

*Adjusted for age, gender and total energy intake

**Additionally adjusted for marital status, smoking, education, j*ob status*, salt intake, multi-vitamin supplement use

***Further adjustment for BMI

## Discussion

We aimed to cross-sectionally investigate the relationship between DII and DIL with indices of mental health. The results showed no significant relation between DIL and DII with odds of depression or anxiety in crude and adjusted models; however, higher DIL was associated with lower odds of stress.

To our knowledge there is only one previous study that investigated the association between DII and DIL and psychological disorders. Anjom-Shoae et al. [22]*,* conducted a cross-sectional study in Isfahan, Iran, that also found no association between DIL and DII with odds of anxiety or depression in the whole population. Although, in contrast to our results, they found that women in the higher quartiles of DII/DIL had higher depression scores. This was not a strong finding as regression analysis of depression as a continuous variable showed no significant linear association between DIL and DII and depression in both sexes. This disagreement of results might also be due to the use of different questionnaires for measuring dietary intake and psychological disorders. In Anjom-Shoae et al. study dietary intake were assessed via a 106-item semi-quantitative FFQ (DS-FFQ) and anxiety and depression were measured by the Iranian validated version of Hospital Anxiety and Depression Scale, while in this study 178-items FFQ and Dass 21 questionnaires were used for assessing dietary intake and psychological disorders respectively. It should be noted that our larger sample size (n = 7384 compared to 3172) is probably more representative for the general population.

Obesity has negative effects on mental health through different mechanisms. First, releasing proinflammatory cytokines such as Tumour Necrosis Factor alpha (TNF-α) and interleukin-6 (IL-6) from hypertrophic adipose tissue, is accompanied by an inflammatory response [20]. Consequently, inflammatory cytokines increase in the circulation and even reach the central nervous system; here they interfere with the hypothalamus–pituitary-adrenal (HPA) axis leading to neuroinflammation [21]. Furthermore, indoleamine 2,3-dioxygenase (IDO) is an enzyme which prevents production of serotonin by catabolizing tryptophan into kynurenine [31]. Increased activity of IDO and reduction in tryptophan serum levels have been reported in obese patients [32]. At least, two studies have investigated the association between DII and DIL and obesity. Adherence to a diet with a high DIL was associated with greater odds of general obesity in women in one study [17] but not supported in another [33]. The BMI for participants of this study did not differ among different quartiles of DII and DIL, which can partly explain the null findings between DII and DIL and psychological disorders.

Although data on DII and DIL are limited, several studies assessed the relationship between GI and GL with psychological disorders [34–37]. A systematic review and meta-analysis showed no significant association between either dietary GI or GL and odds of depression in cross-sectional studies, which partially agrees with our data as most of the food with higher GI and GL also have higher DII and DIL respectively [14]. However, a significant positive relation was observed between dietary GI and depression in clinical trials and cohort studies [38].

Confirming the findings of a previous study [22], our results revealed no significant link between DII or DIL and anxiety. However, we found an inverse association between DIL and stress score. Only one previous study investigated this hypothesis, which reported no significant relationship between DII or DIL and psychological distress [22]. As mentioned above, using different questionnaires and our use of a larger sample size may explain this inconsistency. Moreover, another study showed that higher dietary GL is associated with a lower odds ratio of psychological distress [34]. It is well stablished that foods with higher GL give rise to more insulin secretion and thus a higher IL. Insulin release following consumption of carbohydrate-rich foods leads to elevated plasma tryptophan levels and is associated with increased synthesis of serotonin, a neurotransmitter involved in mental function [39]. Furthermore, stress is controlled by the HPA axis. It has been shown that consumption of sugar-rich foods reduces the feeling of stress by decreasing the HPA axis [40, 41]. The participants of the highest quartiles of DIL of this study consumed more sugars, which might lead them to experience lower levels of stress. However, controlling stress in this way is temporary, and regular consumption of these foods can lead to obesity [41]. In our analysis, the relationship between DIL and stress remained after adjustment for BMI; however, stress correlates with visceral obesity [42]. Further research with a nuanced approach to body composition measurement is warranted.

The present study has several limitations. First, we cannot infer causality due to the cross-sectional design of the study. Second, no biochemical indices were measured in our study, which limits the detection of participants with chronic disease. Third, the II of some food items were not available in the food lists of the previous studies thus, we used the similar food items as substitutes. On the other hand, our studies’ strengths included a large sample size and controlled for potential confounders such as supplements and dietary intake.

In conclusion, consumption of foods with higher II as well as IL was associated with lower stress; however, no significant relation was observed between II or IL and risk of depression and anxiety. Further studies with longitudinal design are needed to confirm these results.

## Supplementary Information

**Additional file 1**.** Table S1**. General characteristics of study participants [Mean ± SD or % (n) & OR (CI)] for presence of psychological disturbances.

**Additional file 2**.** Table S2**. Additional details about Dietary intakes of study participants across quartiles of dietary insulin index and load.

## Data Availability

The data and materials of the current study are available from the corresponding author on reasonable request.
