# Supplementary material for: The association between dietary insulin index and load with mental health
Source: BMC Psychol. 2022 Sep 19;10:218. doi: 10.1186/s40359-022-00925-2 (PMC9483254; doi:10.1186/s40359-022-00925-2)
Supplement: Supplementary file 1 — Additional file 1. Table S1. General characteristics of study participants [Mean ± SD or % (n) & OR (CI)] for presence of psychological disturbances. [file 40359_2022_925_MOESM1_ESM.doc]

| **Additional file 1: Table S1. General characteristics of study participants [Mean ± SD or % (n) &** **OR (CI)] for presence of psychological disturbances** | | | | | | | |
| --- | --- | --- | --- | --- | --- | --- | --- |
| **Variable** | | **Depression (Yes)** | | **Anxiety (Yes)** | | **Stress (Yes)** | |
|  |  | *Mean ± SD or % (n) ^I^* | *OR (CI) ^II^* | *Mean ± SD or % (n)* | *OR (CI)* | *Mean ± SD or % (n)* | *OR (CI)* |
| **BMI (kg/m2)** | | 27.54±5.49^a^ | 1.02(1_1.03) | 27.44±5.31 | 1.02(1_1.03) | 27.37±5.41 | 1.01(0.98_1.04) |
| **Age (%)** | 20-29 years | 111(19.3)^b^ | 1 | 147(19.6) | 1 | 50(21.2) | 1 |
|  | 30_39 years | 139(24.1) | 1.26(0.93_1.71) | 171(22.8) | 1.17(0.89_1.54) | 59(25) | 1.04(0.67_1.62) |
|  | 40_49 years | 120(20.8) | 0.94(0.67_1.33) | 153(204) | 0.93(0.69_1.26) | 44(18.6) | 0.61(0.37_1.03) |
|  | 50-59 years | 112(19.4) | 0.87(0.61_1.25) | 147(19.6) | 0.90(0.66_1.24) | 43(18.2) | 0.57(0.33V_0.98) |
|  | 60_69 years | 94(16.3) | 0.75(0.51_1.12) | 132(17.6) | 0.89(0.63_1.25) | 40(16.9) | 0.55(0.31_0.98) |
| **Marriage (%)** | Single | 89(15.6) | 1 | 112(15.1) | 1 | 36(15.3) | 1 |
|  | Married | 450(78.9) | 0.50(0.36_0.68) | 593(79.8) | 0.51(0.38_0.68) | 189(80.4) | 0.50(0.31_0.80) |
|  | Widowed or Divorced | 31(5.4) | 0.85(0.50_1.46) | 38(5.1) | 0.83(0.50_1.35) | 10(4.3) | 0.59(0.25_1.37) |
| **Smoking status (%)** | Never smoker | 462(82.2) | 1 | 593(80.9) | 1 | 186(80.5) | 1 |
|  | Current smoker | 85(15.1) | 1.82(1.37_2.41) | 121(16.5) | 1.99(1.56_2.53) | 43(18.6) | 2.51(1.67_3.78) |
|  | Ex_smoker | 15(2.7) | 2.17(1.21_3.90) | 19(2.6) | 2.11(1.25_3.59) | 2(0.9) | 0.86(0.20_3.57) |
| **Gender, male (%)** | | 272(47.1) | 0.94(0.73_1.22) | 377(50) | 0.86(0.68_1.09) | 102(42.9) | 1.27(0.52_1.92) |
| **Job (%)** | Unemployed | 112(20) | 1 | 150(20.5) | 1 | 39(16.9) | 1 |
|  | Government employee | 294(52.4) | 1.17 (0.88_1.55) | 363(49.7) | 1.19(0.92_1.52) | 128(55.4) | 1.27(0.82_1.97) |
|  | Manual worker | 23(4.1) | 1.17(0.71_1.92) | 31(4.2) | 1.23(0.79_1.91) | 13(5.6) | 1.75(0.88_3.48) |
|  | Freelance job | 132(23.5) | 0.76(0.57_1.01) | 186(25.5) | 0.81(0.63_1.04) | 51(22.1) | 0.78(0.49_1.24) |
| **Education (%)** | Illiterate | 173(30.1) | 1 | 217(29) | 1 | 83(35.2) | 1 |
|  | Middle school | 179(31.2) | 0.76(059_0.97) | 242(32.3) | 0.87(0.69_1.09) | 73(30.9) | 0.57(0.39_0.83) |
|  | diploma | 152(26.5) | 0.57(0.43_0.75) | 207(27.6) | 0.65(0.51_0.83) | 60(25.4) | 0.40(0.26_0.60) |
|  | Bachelor’s degree | 58(10.1) | 0.47(0.33_0.68) | 69(9.2) | 0.47(0.33_0.65) | 20(8.5) | 0.27(0.15_0.49) |
|  | Master and doctor | 12(2.1) | 0.46(0.24_0.88) | 14(1.9) | 0.46(0.25_0.83) | 0 | 0 |
| **Multi*-*vitamins supplement (%)** | Never | 479(83.9) | 1 | 627(84) | 1 | 201(85.2) | 1 |
|  | 1-3/month | 42(7.4) | 1.35(0.93_1.95) | 46(6.2) | 1.05(0.74_1.49) | 13(5.5) | 0.84(0.44_1.61) |
|  | Minimal once a week | 50(8.8) | 1.38(0.99_1.91) | 73(9.8) | 1.56(1.18_2.06) | 22(9.3) | 1.23(0.75_2.02) |
| **Energy intake (kcal)** | | 2803.16±1384.20 | 1.01(0.98_1.04) | 2851.64±1399.73 | 1 (1_1) | 2791.56±1425.27 | 1 (1_1) |
| ^I^ Obtained from χ2 test and one-way Anova for categorical and continuous variables, respectively.  ^II^ Obtained from logistic regression | | | | | | | |
