# Supplementary material for: The association between dietary insulin index and load with mental health
Source: BMC Psychol. 2022 Sep 19;10:218. doi: 10.1186/s40359-022-00925-2 (PMC9483254; doi:10.1186/s40359-022-00925-2)
Supplement: Supplementary file 2 — Additional file 2. Table S2. Additional details about Dietary intakes of study participants across quartiles of dietary insulin index and load. [file 40359_2022_925_MOESM2_ESM.docx]

| **Additional file 2: Table S2. Additional details about Dietary intakes of study participants across quartiles of dietary insulin index and load**  ^I^ | | | | | | |
| --- | --- | --- | --- | --- | --- | --- |
| F DII | df DII | | F DIL | df DIL | | **Nutrients** |
| 53.89 | 3 | Between Groups | 9587.825 | 3 | Between Groups | Energy intake (kcal) |
|  | 7570 | Within Groups |  | 7570 | Within Groups |  |
|  | 7573 | Total |  | 7573 | Total |  |
| 199.20 | 3 | Between Groups | 1935.888 | 3 | Between Groups | Protein  (% of total daily energy) |
|  | 7570 | Within Groups |  | 7570 | Within Groups |  |
|  | 7573 | Total |  | 7573 | Total |  |
| 51.23 | 3 | Between Groups | 7175.829 | 3 | Between Groups | Carbohydrate  (% of total daily energy) |
|  | 7570 | Within Groups |  | 7570 | Within Groups |  |
|  | 7573 | Total |  | 7573 | Total |  |
| 187.40 | 3 | Between Groups | 2535.625 | 3 | Between Groups | Fat  (% of total daily energy) |
|  | 7570 | Within Groups |  | 7570 | Within Groups |  |
|  |  |  |  | 7573 | Total |  |
|  | 7573 | Total |  |  |  |  |
| 108.07 | 3 | Between Groups | 294.825 | 3 | Between Groups | Cholesterol (mg) |
|  | 7570 | Within Groups |  | 7570 | Within Groups |  |
|  | 7573 | Total |  | 7573 | Total |  |
| 195.03 | 3 | Between Groups | 1905.175 | 3 | Between Groups | SFA (g) |
|  | 7570 | Within Groups |  | 7570 | Within Groups |  |
|  | 7573 | Total |  | 7573 | Total |  |
| 108.05 | 3 | Between Groups | 249.959 | 3 | Between Groups | Vitamin E (mg/day) |
|  | 7570 | Within Groups |  | 7570 | Within Groups |  |
|  | 7573 | Total |  | 7573 | Total |  |
| 195.03 | 3 | Between Groups | 554.676 | 3 | Between Groups | Vitamin C (mg) |
|  | 7570 | Within Groups |  | 7570 | Within Groups |  |
|  | 7573 | Total |  | 7573 | Total |  |
| 168.07 | 3 | Between Groups | 1454.402 | 3 | Between Groups | Folic acid (µg) |
|  | 7570 | Within Groups |  | 7570 | Within Groups |  |
|  | 7573 | Total |  | 7573 | Total |  |
| 14.90 | 3 | Between Groups | 2998.577 | 3 | Between Groups | Magnesium (mg) |
|  | 7570 | Within Groups |  | 7570 | Within Groups |  |
|  | 7573 | Total |  | 7573 | Total |  |
| 97.26 | 3 | Between Groups | 47.361 | 3 | Between Groups | Fruits |
|  | 7570 | Within Groups |  | 7570 | Within Groups |  |
|  | 7573 | Total |  | 7573 | Total |  |
| 74.81 | 3 | Between Groups | 135.90 | 3 | Between Groups | Vegetables |
|  | 7570 | Within Groups |  | 7570 | Within Groups |  |
|  | 7573 | Total |  | 7573 | Total |  |
| 18.10 | 3 | Between Groups | 91.99 | 3 | Between Groups | Red meat |
|  | 7570 | Within Groups |  | 7570 | Within Groups |  |
|  | 7573 | Total |  | 7573 | Total |  |
| 39.83 | 3 | Between Groups | 21.39 | 3 | Between Groups | Fish |
|  | 7570 | Within Groups |  | 7570 | Within Groups |  |
|  | 7573 | Total |  | 7573 | Total |  |
| 14.87 | 3 | Between Groups | 99.91 | 3 | Between Groups | Dairy |
|  | 7570 | Within Groups |  | 7570 | Within Groups |  |
|  | 7573 | Total |  | 7573 | Total |  |
| 17.20 | 3 | Between Groups | 14.07 | 3 | Between Groups | Legumes and nuts |
|  | 7570 | Within Groups |  |  |  |  |
|  | 7573 | Total |  | 7570 | Within Groups |  |
|  |  |  |  | 7573 | Total |  |
| 53.87 | 3 | Between Groups | 206.26 | 3 | Between Groups | Whole grains |
|  | 7570 | Within Groups |  | 7570 | Within Groups |  |
|  | 7573 | Total |  | 7573 | Total |  |
| 46.54 | 3 | Between Groups | 249.87 | 3 | Between Groups | Refined grains |
|  | 7570 | Within Groups |  | 7570 | Within Groups |  |
|  | 7573 | Total |  | 7573 | Total |  |
| 576.47 | 3 | Between Groups | 5.05 | 3 | Between Groups | Sugars |
|  | 7570 | Within Groups |  | 7570 | Within Groups |  |
|  | 7573 | Total |  | 7573 | Total |  |
| 12.73 | 3 | Between Groups | 186.54 | 3 | Between Groups | Salt |
|  | 7570 | Within Groups |  | 7570 | Within Groups |  |
|  | 7573 | Total |  | 7573 | Total |  |
| DII, Dietary insulin index; DIL, Dietary insulin load  ^I^ Obtained from one-way Anova | | | | | | |
